# High content phenotypic screening identifies serotonin receptor modulators with selective activity upon breast cancer cell cycle and cytokine signaling pathways

**DOI:** 10.1016/j.bmc.2019.115209

**Published:** 2020-01-01

**Authors:** Scott J. Warchal, John C. Dawson, Emelie Shepherd, Alison F. Munro, Rebecca E. Hughes, Ashraff Makda, Neil O. Carragher

**Affiliations:** Cancer Research UK Edinburgh Centre, MRC Institute of Genetics and Molecular Medicine, University of Edinburgh, EH4 2XR Edinburgh, United Kingdom

**Keywords:** High content imaging, Cell Painting, Phenotypic screening, Serotonin, Triflupromazine, Pathway analysis, Breast cancer, Pharmacogenomics

## Abstract

Heterogeneity in disease mechanisms between genetically distinct patients contributes to high attrition rates in late stage clinical drug development. New personalized medicine strategies aim to identify predictive biomarkers which stratify patients most likely to respond to a particular therapy. However, for complex multifactorial diseases not characterized by a single genetic driver, empirical approaches to identifying predictive biomarkers and the most promising therapies for personalized medicine are required. *In vitro* pharmacogenomics seeks to correlate *in vitro* drug sensitivity testing across panels of genetically distinct cell models with genomic, gene expression or proteomic data to identify predictive biomarkers of drug response. However, the vast majority of *in vitro* pharmacogenomic studies performed to date are limited to dose-response screening upon a single viability assay endpoint. In this article we describe the application of multiparametric high content phenotypic screening and the theta comparative cell scoring method to quantify and rank compound hits, screened at a single concentration, which induce a broad variety of divergent phenotypic responses between distinct breast cancer cell lines. High content screening followed by transcriptomic pathway analysis identified serotonin receptor modulators which display selective activity upon breast cancer cell cycle and cytokine signaling pathways correlating with inhibition of cell growth and survival. These methods describe a new evidence-led approach to rapidly identify compounds which display distinct response between different cell types. The results presented also warrant further investigation of the selective activity of serotonin receptor modulators upon breast cancer cell growth and survival as a potential drug repurposing opportunity.

## Graphical abstract

**Figure:**
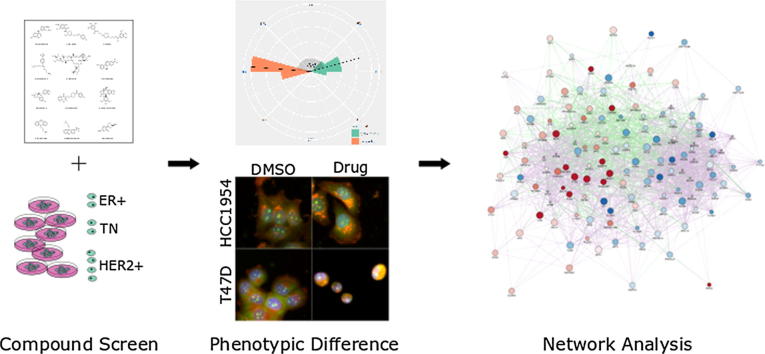

## 1 Introduction

For many complex diseases, heterogeneity in the molecular mechanisms of disease onset and progression between distinct patients contributes to high attrition in clinical drug development. Advances in next generation sequencing (NGS) and classification of patients into molecularly defined subgroups support personalized medicine strategies, which utilize predictive biomarkers to identify patient subgroups, which are most likely to respond to a specific therapy.^1^, ^2^ In cancer, highly selective drugs targeted at genetically defined clinical subtypes has demonstrated significant success where drug mechanism-of-action (MOA) can be directly mapped to amplifications or mutations of specific therapeutic targets or to key vulnerabilities such as DNA repair defects.^3^, ^4^, ^5^, ^6^ However, for the majority of patients, the underlying molecular drivers of disease are either unknown or complicated by multiple genetic aberrations and redundant pathways confounding the identification of the most promising therapeutic targets, candidate drugs and biomarker strategies.

Recent advances of *in vitro* cell based assay screening technologies that enable rapid screening of large numbers of approved drugs, experimental drugs and diverse chemical libraries across panels of genetically distinct cell lines combined with genetic and proteomic profiling are well placed to support more unbiased evidence-led preclinical approaches to personalized medicine discovery.^7^, ^8^ Advances in new cell based assay technologies including automated high content imaging and molecular cell profiling technologies (e.g. NGS and miniaturized array based transcriptomic and proteomics) present new opportunities for incorporating more relevant and informative models into drug discovery.^9^ For example, the adaptation of patient-derived primary cell samples for high throughput screening have supported the application of drug sensitivity and resistance testing (DSRT) to provide a more patient-centric approach to drug discovery and development.^10^ In a typical DSRT assay, cancer cells taken directly from patients are purified and placed in multi-well plates for screening of several hundred clinically approved or experimental cancer drugs at multiple concentrations and cell viability is measured after 72 h (for example references^10^, ^11^, ^12^, ^13^). In leukemia where the *ex vivo* material (for example, liquid biopsy samples) are more readily available for drug testing than in solid tumors, patient-derived samples have recently been utilized for potential drug repositioning^10^, ^14^ and combined with molecular profiling to identify biomarkers for personalized acute myeloid leukemia (AML) therapy.^10^

*In vitro* pharmacogenomics describes the application of compound screening across genetically distinct cell types and correlation of drug sensitivity with genomic and gene expression datasets to elucidate drug mechanism of action and identify biomarkers of response.^15^, ^16^ Multiple articles have described the application of high throughput *in vitro* pharmacogenomics across genetically distinct panels of cancer cell lines and large databases linking gene expression data and drug sensitivity have been developed.^17^, ^18^ However, the majority of DSRT and *in vitro* pharmacogenomic studies performed to date have used single cell viability endpoints, which include application to complex models and/or patient biopsies.^19^ However, such single viability endpoints preclude more detailed phenotypic response analysis of complex and diverse co-culture, 3D cell models or other phenotypic endpoints which may further inform clinical applications (e.g. cell motility, autophagy, DNA damage/repair defects and heterogeneity at single cell level).

The integration of automated high-throughput microscope platforms with the latest advances in multiparametric image analysis, multivariate statistics, machine learning and new computational biology resources enable more sophisticated classification of cell phenotypes across cell based assay systems at scale. These advances support the new disciplines of high content analysis and phenotypic profiling which compare similarities and dissimilarities between drug MOA across cell based assays.^20^, ^21^, ^22^, ^23^, ^24^ It is anticipated that further development of these methods will better inform target identification, hit identification and hit-to-lead medicinal chemistry programs in relevant models of disease. Further integration of high content phenotypic profiling data across well characterized patient-derived cells, and established cell line panels with genomics, proteomics and computational biology approaches are also well placed to further advance *in vitro* pharmacogenomics and drug repurposing studies.

We recently described the application of a method, Theta Comparative Cell Scoring (TCCS), which uses multiparametric high content imaging data to quantify how similar or dissimilar a compound induced phenotype is between genetically distinct cell types.^25^, ^26^ We proposed that the TCCS method will support the application of *in vitro* pharmacogenomic and drug repurposing studies beyond simple univariate assay endpoints towards more complex assays, phenotypic endpoints and novel therapeutic classes. Here, we describe the application of a high content Cell Painting assay and the TCCS method to a 1280 compound FDA-approved small molecule library screen across a panel of 8 genetically distinct human breast cancer cell lines. We used the TCCS method to rank compounds which display the most divergent phenotypic response across the breast cancer cell panel. We identified a series of 4 known serotonin receptor modulators (fluvoxamine, cisapride, protriptyline and triflupromazine) which rank among the top 12 compounds promoting the most distinct phenotypic response between breast cancer cell line pairs. Transcriptomic pathway analysis performed on the most potent serotonin modulator (triflupromazine) demonstrates selective down regulation of multiple cell cycle pathways and upregulation of tumor necrosis factor (TNF) signaling pathways in the most sensitive breast cancer cell type. Thus, combined high content phenotypic screening, and pathway network analysis identifies an existing serotonin receptor modulator with selective activity upon breast cancer cell cycle and TNF signaling pathways representing a potential drug repurposing opportunity for specific breast cancer types.

## 2 Results and discussion

**Fig. 1:**
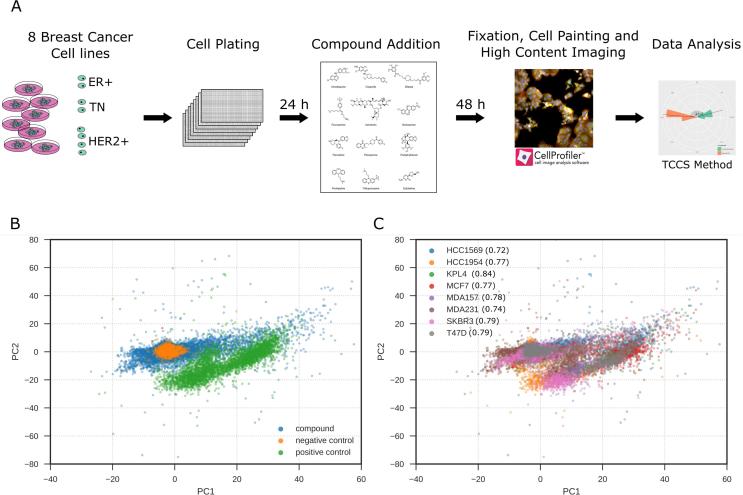
Summary of phenotypic screen. (A) Schematic of screening strategy. (B) Principal component analysis (PCA) of phenotypic screen. Data points color coded: drug treatment, positive control (300 nM staurosporine) or negative control (0.1% DMSO). (C) PCA analysis of phenotypic screen color coded by cell line. Multivariate Z’-factor analysis between negative (0.1% DMSO) and positive (0.3 μM staurosporine) controls by cell line is indicated in brackets.

The Prestwick Chemical Library of 1280 FDA-approved compounds at a single concentration of 1 μM was profiled in a phenotypic high-content image based assay across eight breast cancer cell-lines (HCC1569; HCC1954; KPL4; MCF7; MDA-MB-157; MDA-MB-231; SKBR3; T47D) which are classified into three clinical subtypes; estrogen receptor (ER) positive, HER2 amplified or triple negative (TN) for ER, progesterone receptor and HER2 (Fig. 1A).

**Fig. 2:**
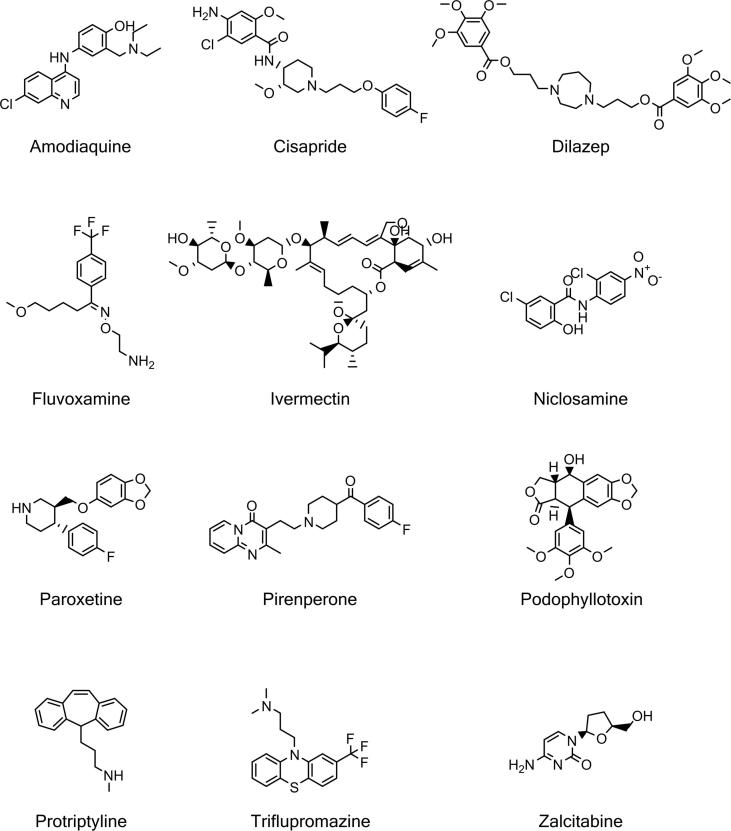
Chemical structures of hit compounds that are phenotypically different between cell line pairs.

We sought to identify compounds that induced distinct phenotypic responses between cell lines using a Cell Painting assay as an unbiased phenotypic profiling method.^21^ We quantified multiple morphological features from the images using CellProfiler image analysis software^27^ and aggregated single cell data to an image median. Each cell line has different genetic backgrounds^28^ and such changes can drive phenotypic differences between each cell line, for example, loss of E-cadherin is a hallmark of epithelial to mesenchymal transition as it is a critical component of epithelial cell-cell junctions^29^ and is often differentially expressed between cell lines resulting in significant morphological differences. Therefore, to allow comparison of cellular phenotypes across the panel of cell lines, compound treated cell feature data was normalized and standardized to the plate negative control values and visualized using principal component analysis to reveal a clear separation between the positive (300 nM staurosporine) and negative control (0.1% DMSO) treatments (Fig. 1B and C). This step effectively removed the variation in the basal cell line morphologies (Supplementary Fig. S1). This allowed us to compare the effect of compound treatment on the phenotypic response across a morphologically distinct cell-line panel (Fig. 1A). Assay robustness was confirmed by using a multivariate Z’ factor analysis between negative and positive controls^30^ giving a score of 0.6 for the pooled cell-lines, and greater than 0.7 for individual cell-lines (Fig. 1A) demonstrating a robust screening assay. For each phenotypically active compound in the Prestwick FDA-approved library (Table 1), the difference in response between pairs of cell-lines was measured using the TCCS method,^26^ and a rank product of the delta theta score was used to list compound-cell line pairs in terms of the greatest differential effects; the 266 compound-cell line pairs are shown in Supplementary Table S1. Compounds that demonstrated distinct phenotypic responses between cell lines were confirmed by replication studies and triaged by selecting those with interesting MOAs, for example by removing known anti-cancer cytotoxic compounds, including several microtubule disruptors: twelve hits were selected for further study (Fig. 2 and Table 2).

**Table 1:** Number of active compounds in the Prestwick library per cell-line.

Cell line	# active compounds
HCC1569	283
HCC1954	182
KPL4	236
MCF7	287
MDA-MB-157	96
MDA-MB-231	352
SKBR3	218
T47D	327

**Table 2:** Hits selected from the Prestwick library which produced distinct phenotypic responses between cell-lines. SERT: serotonin reuptake transporter, SSRI: selective serotonin reuptake inhibitor, 5-HT: 5-hydroxytryptamine, D1/2 dopamine receptor.

Compound	Usage/MoA
Amodiaquine	Anti-malarial
Cisapride	5-HT4 agonist
Dilazep	Vasodilator. Adenosine reuptake inhibitor
Fluvoxamine	Anti-depressant. SSRI
Ivermectin	Anti-helmintic. GluCl agonist
Niclosamide	Anti-helmintic
Paroxetine	Anti-depressant. SSRI
Pirenperone	5-HT2A antagonist
Podophyllotoxin	Microtubule destabiliser
Protriptyline	Tricyclic anti-depressant. NA, SERT
Triflupromazine	Antipsychotic. D1, D2 antagonist
Zalcitabine	Nucleoside reverse transcriptase inhibitor

**Fig. 3:**
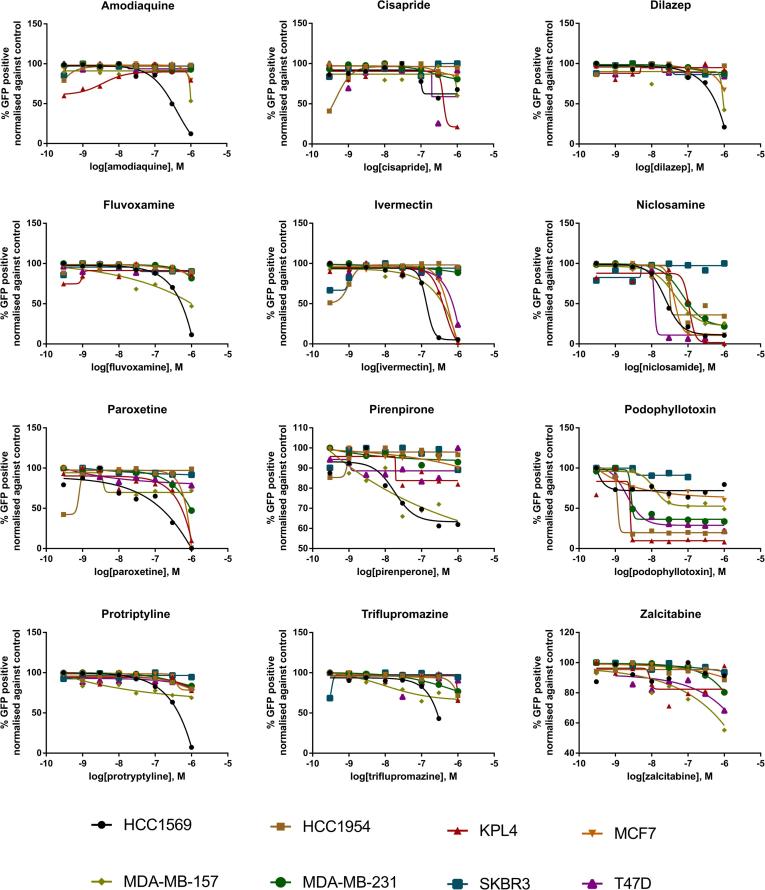
Concentration-response curves for 12 hits from the Prestwick Chemical Library. Compounds were used in a 2D cell proliferation assay measuring cell count expressed as the percentage of the DMSO control after 72 h. Note: compounds were originally screened at a concentration of 1 μM using the Cell Painting assay.

Using GFP-NucLight expressing breast cancer cells, selected hits were tested as an 8 point semi-log range of concentrations (0.3 nM to 1 µM) and cell proliferation was monitored over 72 h in the IncuCyte ZOOM microscope. GFP cell-count normalized to the DMSO negative control was used as a measure of cell proliferation over 72 h (Fig. 3) and despite a selection criteria aiming to limit overtly cytotoxic compounds, 11 out of the 12 compounds demonstrated a reduction in cell-count in at least one of the cell-lines at the higher concentrations tested. The HCC1569 cell-line demonstrated the greatest sensitivity to the majority of the tested compounds while podophyllotoxin proved to be especially potent with a relative cell-count below 50% at the lowest tested concentration of 0.3 nM in 5 of the cell-lines. Interestingly, the cell proliferation assay did not readily discern differences between protriptyline and triflupromazine which induced a distinct phenotypic response between cell lines as determined by the cell painting assay with compound concentrations up to 1 μM (Fig. 3 and Table 2). These results suggest that the Cell Painting assay provides a more sensitive and rapid readout for detection of cell growth and viability phenotypes at sub-lethal concentrations of drug and/or prior to overt growth inhibition or cytotoxicity effects. Furthermore, the added phenotypic information pertaining to drug MOA including effects on cell cycle, cytokinesis and cytoskeletal morphology elucidated by the high content Cell Painting assay provides added value relative to univariate cell growth and cytotoxicity analysis. Other advantages of high content Cell Painting and TCCS analysis for *in vitro* pharmacogenomic studies over traditional cell proliferation and viability assays include: Speed (48 h phenotypic assay relative to long-term 3-, 5- or 7-day viability or growth assays); Single-point concentration testing relative to dose-response testing substantially influencing throughput; Suitability for discriminating phenotypic response in heterogeneous cell subpopulations or co-culture assays. Applicable to any assay or endpoint that can be discerned by image-based phenotypic analysis including disease models and therapeutic classes not defined by cell growth or viability.

Of the top 15 compound induced-phenotypic differences between cell lines determined by Cell Painting and TCCS analysis, half involved HCC1954 cells. Among the top 12 FDA-approved drugs which promote distinct phenotypic response between cell lines are 4 structurally distinct small molecule drugs with previously reported serotonin modulator activity; fluvoxamine, cisapride, protriptyline and triflupromazine. Fluvoxamine (DrugBank #DB00176) is an approved antidepressant which functions pharmacologically as a selective serotonin reuptake inhibitor. Fluvoxamine blocks the reuptake of serotonin at the serotonin reuptake pump of the neuronal membrane, enhancing the actions of serotonin on 5HT1A autoreceptors. Studies have also demonstrated that fluvoxamine has virtually no affinity for α1- or α2-adrenergic, β-adrenergic, muscarinic, dopamine D2, histamine H1, GABA-benzodiazepine, opiate, 5-HT1, or 5-HT2 receptors. Cisapride (DrugBank #DB00604) was approved as a treatment for heartburn due to gastroesophageal reflux disease. Cisapride acts through the stimulation of the serotonin 5-hydroxytryptamine (5-HT4, 5-HT3A, 5-HT2A) receptors which increases acetylcholine release in the enteric nervous system. Cisapride does not induce muscarinic or nicotinic receptor stimulation, nor does it inhibit acetylcholinesterase activity. Triflupromazine (DrugBank #DB00508) is a member of a class of drugs called phenothiazine’s and is used to treat schizophrenia and other psychotic disorders. Triflupromazine binds to the dopamine D1 and dopamine D2 receptors and inhibits their activity. Triflupromazine also binds the muscarinic acetylcholine receptors (M1 and M2) and the serotonin receptor 5-HT2. Protriptyline (DrugBank #DB00344) is a dibenzocycloheptene-derivative tricyclic antidepressant (TCA). TCAs are structurally similar to phenothiazines and are potent inhibitors of serotonin and norepinephrine reuptake and also block histamine H1 receptors, α1-adrenergic receptors and muscarinic receptors.

**Fig. 4:**
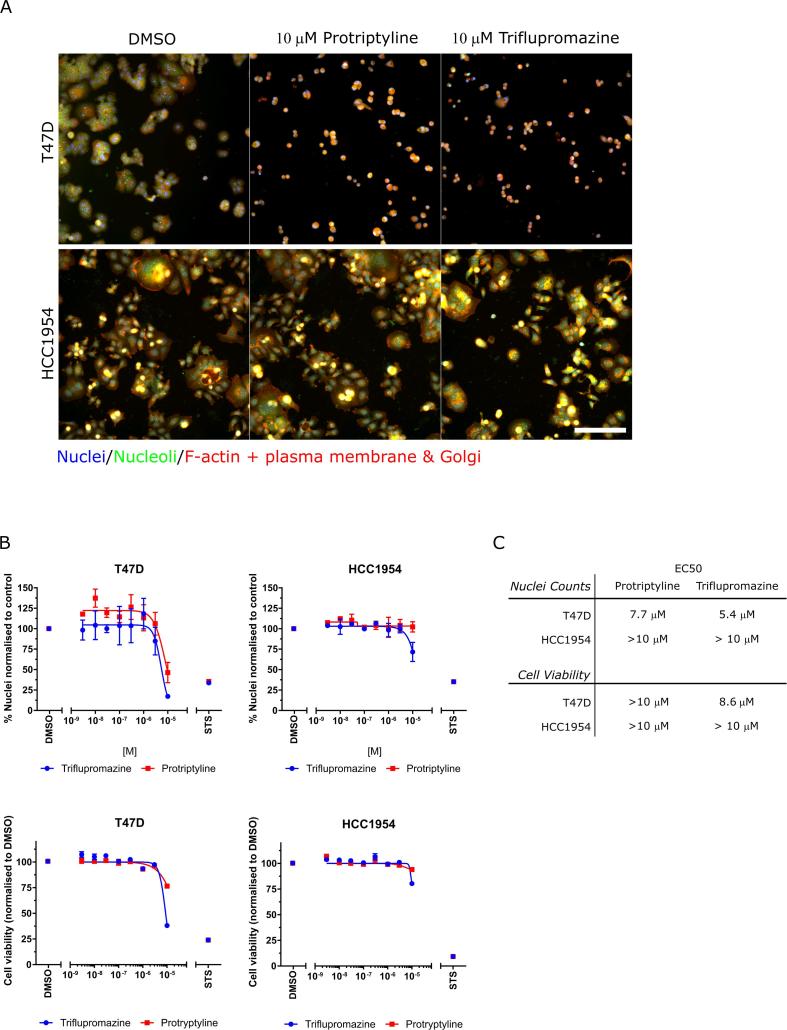
Effects of protriptyline and triflupromazine using nuclei/cell counting and viability assays in T47D and HCC1954 cells. (A) Cell lines treated with protriptyline or triflupromazine for 48 h. Scale bar is 100 μm. (B) Quantification of nuclear counts (top) after 48 h and cell viability (bottom) after 72 h of compound treatment. n = 3, mean ± SEM is shown. STS, staurosporine (300 nM). (C) Table of EC50 values for growth inhibition.

Serotonin has recently emerged as a growth factor for several human tumor cell types and the pattern of serotonin receptor subtype expression becomes dysregulated in several human tumors when compared with normal cells.^31^ The serotonin-induced signaling pathways that promote tumor progression are however complex and only partly understood, however serotonin receptors are expressed in breast cancer cells and tissue^32^, ^33^, ^34^ and serotonin receptor expression correlates with estrogen and HER2 receptor expression.^34^ Further, analysis of the target genes for protriptyline (*SLC6A2* and *SLC6A4*) and triflupromazine (*HTR2B*, *CHRM1*, *CHRM2*, *DRD1*, and *DRD2*) as well as their wider family members using RNAseq data from the Cancer Cell Line Encyclopedia (Supplementary Fig. S2) demonstrated expression of *CHRM1*, *DRD2*, *HTR2B*, *SLC6A2* and *SLC6A4* in 54 breast cancer cells, including the cancer cell lines we have used in our study. Therefore, we selected two serotonin targeting hit compounds, protriptyline and triflupromazine to study in more detail in the HCC1954 cells and T47D cells. Protryptyline and triflupromazine both induced a similar phenotypic difference between HCC1954 and T47D cells in the Cell Painting assay at 1 μM, a concentration that did not reduce cell number. Changes in cell morphology became more pronounced when the concentration range was expanded up to 10 μM (Fig. 4A), with the T47D cells displaying a reduced number of cells and morphological changes in response to compound treatment which was not observed in HCC1954 cells. Furthermore, T47D cells were more sensitive to growth inhibition following protriptyline or triflupromazine treatment than the HCC1954 cells, as assessed by quantifying nuclei counts and cell viability after 48 h treatment (Fig. 4B and C). Taken together, this data demonstrates that cellular phenotypic profiling using the Cell Painting assay, at sub-toxic/cytostatic compound concentrations, can be used to predict toxic/cytostatic compound effects observed using a broader range of concentrations in a cell viability/cell count assay.

**Fig. 5:**
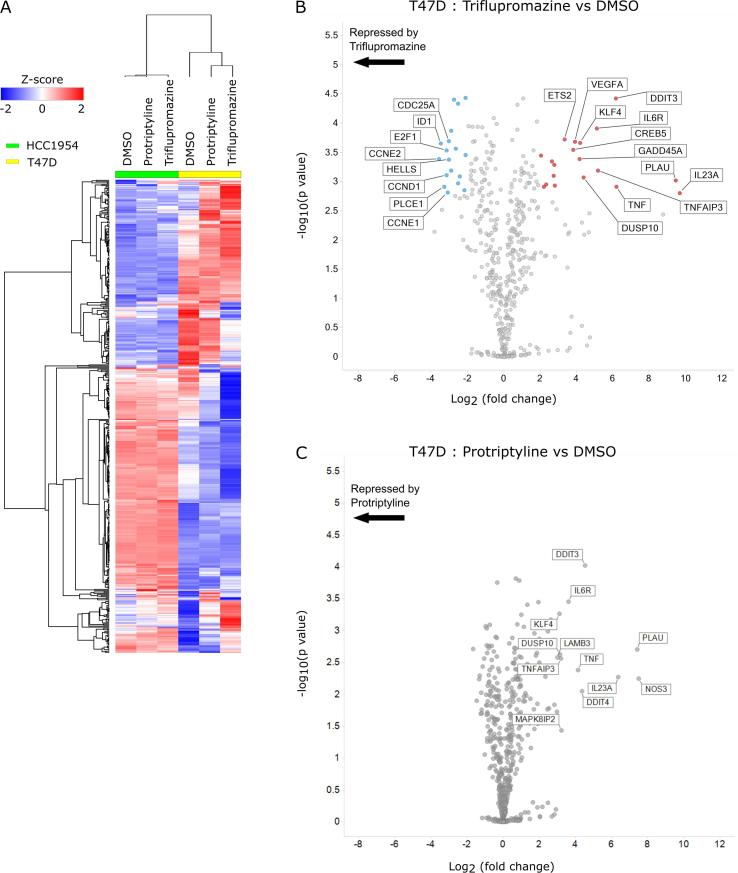
Gene expression analysis of compound treatments in T47D and HCC1954 cell lines. (A) Heatmap of gene expression following 24 h treatment with compound. (B) Differential gene expression analysis of gene changes following triflupromazine treatment for 24 h in the T47D cell line. (C) Differential gene expression analysis of gene changes following protriptyline treatment for 24 h in the T47D cell line. For (B) and (C), genes significantly altered (p < 0.05, Benjamini–Yekutieli-corrected test) are highlighted; up-regulated (red circles) or down-regulated (blue circles). Gene names are displayed for genes with log2(fold change) greater than 3 or less than -3.

**Fig. 6:**
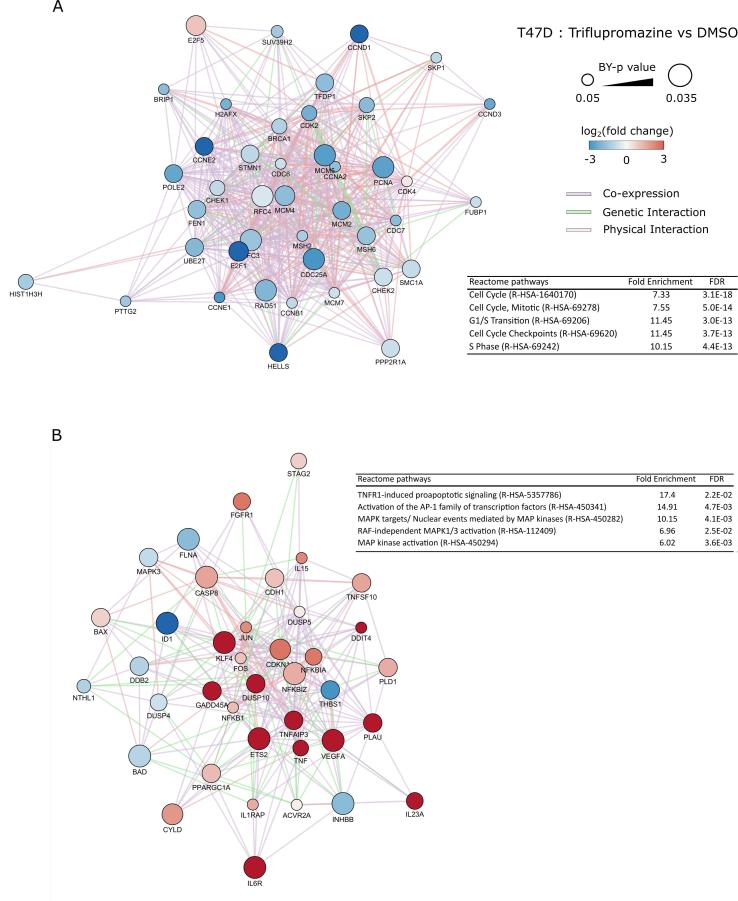
Interaction network analysis of differentially expressed genes in triflupromazine treated T47D cells. (A) Network of cell cycle related genes. (B) Network of TNFR1 signalling genes.

To elucidate the molecular changes in HCC1954 and T47D cells following treatment with protriptyline and triflupromazine, we isolated RNA after 24 h of compound treatment and profiled the changes in gene expression using the Pan-Cancer panel from NanoString consisting of 770 cancer related genes. Gene expression analysis revealed a large number of significantly changed genes following triflupromazine treatment in T47D cells but not HCC1954 cells (Fig. 5A). Differential analysis of drug-induced gene expression changes relative to DMSO in T47D cells revealed a number of significantly up and down regulated genes following treatment with triflupromazine (Fig. 5B and C). Network analysis of significantly (p < 0.05) up- and down-regulated genes following triflupromazine revealed two large connected networks of genes (Fig. 6). A large network of down regulated cell cycle associated genes including core regulators of the cell cycle (Fig. 6A) such as *CCND1* (cyclin D1), *CCNE2* (cyclin E2) and *CCNA2* (cyclin A2). Enrichment analysis of the upregulated gene set revealed activation of the TNFR1 signaling pathway suggesting induction of apoptosis via TNF signaling (Fig. 6B). Interestingly, HCC1954 have a missense point mutation in TRAF2 (Q457L)^18^ located in the receptor binding TRAF-C domain. TRAF2 is a key mediator of TNF receptor signaling. The functional significance of TRAF2 (Q457L) mutation is unclear, however such a missense mutation may impact upon TNF signaling and cell survival signaling during tumor development and response to therapy.^35^ Serotonin is important in mammary gland development, in part produced by the enzyme tryptophan hydroxylase (TPH) expressed by mammary epithelial cells^36^ and TPH1 expression is elevated in tumor cells^34^ (Supplementary Fig. S2).

Up to one-quarter of breast cancer patients suffer clinically significant depression in the year after diagnosis and about half may be prescribed a psychotropic medication, such as a selective serotonin reuptake inhibitor (SSRI), while completing breast cancer therapy. Epidemiology studies on breast cancer recurrence risk related to concurrent use of SSRI antidepressants and tamoxifen indicated breast cancer patients taking SSRIs were at no increased risk of developing breast cancer^37^ or breast cancer recurrence.^38^ Another study highlights SSRIs which inhibit P450 2D6 (CYP2D6), necessary for the bioactivation of tamoxifen, reduce tamoxifen’s effectiveness and contributed to increase mortality when the SSRI paroxetine is used concurrently with tamoxifen.^39^ Our results call for further retrospective epidemiology studies on the use of serotonin receptor modulators which do not inhibit P450 or are administered independent of tamoxifen, however, considerable variation in the prescribing patterns of SSRI antidepressants across the world, with few studies reporting robust data on exact dose or follow‐up regimens may confound this analysis.^40^ In addition, further investigation of serotonin signaling modulation across a broader panel of genetically distinct breast cancers and the interplay with activation of the TNF pathway is needed.

## 3 Conclusions

We have applied a high content Cell Painting assay and the Theta Comparative Cell Scoring (TCCS) method to quantify a similarity score of phenotypic response between genetically distinct breast cancer cell types. TCCS was used to identify four serotonin modulator small molecules (fluvoxamine, cisapride, protriptyline and triflupromazine) which rank among the top 12 FDA approved compounds promoting the most distinct phenotypic response between breast cancer cell lines. NanoString transcriptomic profiling and pathway network analysis was applied to demonstrate that triflupromazine exerts selective activity upon the inhibition of multiple cell cycle pathways and upregulation of TNF receptor signaling in sensitive relative to non-sensitive breast cancer lines. While the current study has made use of a diverse panel of established breast cancer cell lines which may have limited utility in guiding personalized medicine strategies, the methods and results presented in this article demonstrate the value of multiparametric high content phenotypic profiling and pathway network analysis. These methods can also be applied to isogenic and primary patient-derived cell assays composed of genetically distinct cell types to advance phenotypic screening, drug repurposing and future pharmacogenomic analysis across diverse phenotypes.

## 4 Experimental

### 4.1 Cell culture

Cell-lines were all grown in DMEM (#21969-035 Gibco) and supplemented with 10% fetal bovine serum and 2 mM l-glutamine, incubated at 37 °C, humidified and 5% CO2. Optimized cell number per 384 well plate: HCC1569 (1500 cells/well); HCC1954 (1500 cells/well); KPL4 (750 cells/well); MCF7 (1500 cell/well); MDA-MB-231 (750 cell/well); MDA-MB-157 (2000 cells/well); SKBR3 (2000 cells/well); T47D (1500 cell/well). Optimized cell number per 96 well plate: HCC1569 (3000 cells/well); HCC1954 (3000 cells/well); KPL4 (2000 cells/well); MCF7 (3000 cell/well); MDA-MB-231 (2000 cell/well); MDA-MB-157 (3500 cells/well); SKBR3 (3500 cells/well); T47D (3000 cell/well). Cells were seeded in 96- or 384-well optical bottomed imaging plates (#655090 and #781091 Greiner) for image based assays, respectively or standard 96 well plates for all other assays (#655180 Greiner). Plates were incubated for 24 h in a tissue culture incubator before the addition of compounds.

### 4.2 IncuCyte proliferation assay

The lentiviral based IncuCyte® NucLight Reagent (Sartorius) was used to label all breast cancer cell lines with a nuclear-restricted green fluorescent protein (GFP) to enable accurate quantification of cell growth over time. The IncuCyte® platform acquired images and calculated the number of cell nuclei to provide a cell count assay endpoint at sequential time points following cell plating and compound treatment. All analyses presented was performed at 72 h following compound treatment. Note: the morphology and growth rate of the GFP-NucLight labelled cells were confirmed to behave similarly to parental cell lines. NucLight cells were used for the IncuCyte® cell proliferation experiment only, parental cell lines were used for Cell Painting, cell viability and NanoString experiments.

### 4.3 Cell viability assay

For cell viability assays, seeding density for 96-well plate assay format was optimized for each individual cell line to ensure a linear assay range. Cells were seeded in 96 well plates and cultured for 24 h before treatment. Cells were incubated with compounds for 72 h, untreated cells were incubated with 0.1% DMSO (v/v). Alamar Blue (Invitrogen, Paisley, UK) was added (10% v/v) to each well and plates incubated for 3 h. Fluorescence emission was read on an EnVision 2101 multilabel plate reader (PerkinElmer; excitation = 540 nm, emission = 590 nm). All conditions were normalized to plate DMSO control wells.

### 4.4 Prestwick compound library handling

Handling of the Prestwick FDA-approved compound library was performed using a Biomek FX. 1.5 μL from master plates containing 1 mM compound in DMSO was transferred to an intermediate plate containing 74.5 μL of cell culture media for a 1:50 dilution. From the intermediate plate 2.5 μL was transferred to the cell assay plate containing 50 μL volume for a second dilution of 1:20 resulting in a final 1;1000 dilution of compound stock at 0.1%DMSO (v/v) in each assay well. Assay plates were barcoded with cell-line and a sequential number corresponding to the compound source plate.

### 4.5 Cell Painting staining protocol

Cell Painting^21^ and our modified protocol for our panel of breast cancer cell lines is described in.^25^, ^26^ Briefly, cells are fixed by adding an equal volume of 8% paraformaldehyde (#28908 Thermo Scientific) to the existing media resulting in a final paraformaldehyde concentration of 4%, and left to incubate for 30 min at room temperature. The plates are then washed with PBS and permeabilized with 0.1% Triton-X100 solution for 20 min at room temperature. A solution of Cell Painting reagents (Hoeschst 33342; SYTO14; Phalloidin-594: Wheat germ agglutinin-594; Concavalin-488; MitoTracker DeepRed) was made up in 1% bovine serum albumin (BSA) solution. Cell Painting solution was added to plates and incubated for 30 min at room temperature in a dark place. Plates were then washed with PBS three times, and plates were sealed with a plate seal. In a modification to the original Cell Painting assay we add the MitoTracker dye following cell fixation as we found that application of MitoTracker to MDA-MB-231 cells in culture induced morphological changes. We still observe significant signal above noise in a staining pattern resembling mitochondria and we therefore anticipate that post-fixation staining with MitoTracker still provides mechanistic information on mitochondrial number and structure but without any risk of artefactual modulation of cell morphology following live cell exposure.

We selected staurosporine as our phenotypic positive control as it induces a distinctive change in cell morphology across all the breast cancer cell lines at the same concentration (0.3 μM), most likely as a consequence of its broad substrate specificity. This makes staurosporine a very useful control compound for Cell Painting-like morphology analysis across cell line panels, even though there is no clinical application for this compound. Negative (0.1% DMSO) and positive (0.3 μM staurosporine) controls were used for normalization of plate effects and evaluation of the performance of basal cell line normalization of distinct cell morphologies (Supplementary Fig. S1).

### 4.6 Imaging

Imaging was carried out on an ImageXpress micro XL (Molecular Devices, USA) a multi-wavelength wide-field fluorescent microscope equipped with a robotic plate loader (Scara4, PAA, UK). Images were captured in 5 fluorescent channels at 20× magnification, exposure times were kept constant between plates and batches as to not influence intensity values.

### 4.7 Image analysis

Images were analyzed using CellProfiler v2.1.1 to extract morphological features, quantifying 340 morphological features per cell. Briefly, cell nuclei were segmented in the Hoechst stained image based on intensity, clumped nuclei were separated based on shape. Nuclei objects were used as seeds to detect and segment cell-bodies in the cytoplasmic stains of the additional channels. Subcellular structures such as nucleoli and Golgi apparatus were segmented and assigned to parent objects (cells). Using these masks marking the boundary of cellular objects, morphological features are measured for multiple image channels returning per object measurements.

### 4.8 Data analysis

*Pre-processing*: Out of focus and low-quality images were detected through saturation and focus measurements and removed from the dataset. Image averages of single object (cell) measurements were aggregated by taking the median of each measured feature per image. Feature selection was performed by calculating pair-wise correlations of features and removing one of a pair of features that have correlation greater than 0.9, and removing features with very low (<1e^−5^) or zero variance. To normalize the inherent morphological variation between cell lines, features were standardized on a plate-by-plate basis by dividing each feature by the median DMSO response for that feature and scaled by a *z*-score (*z*) to a zero mean and unit variance. Tabular data from CellProfiler measuring morphological features for each cell was aggregated to an image median. Principal components were calculated using the prcomp function in R v3.2, with no centering or scaling as this was performed manually beforehand. The number of principal components to use in the analysis can be determined by specifying beforehand the proportion of variance in the data that should be kept, and then finding the minimum number of principal components that account for that proportion of variance in the dataset.

In order to center the principal component data so that the medoid of the negative control was positioned on the origin, the median value for each feature column for the negative control data was calculated. Then by calculating how much this differs from the origin for each feature, all principal component values were adjusted by this difference. Inactive compounds were identified by determining a minimum cut-off distance to the negative control centroid in principal component space. This was calculated by first finding the l1 norm from each compound to the negative control centroid. The standard deviation of all these distances was calculated and any compound which was within 2 standard deviations of the negative control centroid was deemed inactive, if a compound was found to be inactive in any one of the eight cell lines it was removed from the analysis.

*Phenotypic characterization:* Following data pre-processing, distinct phenotypic responses between active compounds were calculated using the TCCS method.^25^, ^26^ An initial hit list was created by ranking compounds and cell-line pairs by decreasing Δθ. Compounds were triaged by removing those with less interesting mechanistic properties such as microtubule disruptors leaving 14 hits. From these 14 hits, 2 were not easily available due to lack of a commercial supplier (pinaverium bromide) or being a controlled substance (3,4-dimethoxyphenethylamine). For each hit compound Δθ values were calculated between all pairs of cell-lines for each, and ranked by order of decreasing Δθ, so that compound-cell-line-pairs with a more distinct phenotypic response received a lower rank. A rank product^41^ was calculated from the replicates and compound-cell-line-pairs were sorted by increasing rank product.

### 4.9 RNA isolation and NanoString analysis

T47D and HCC1954 cells were seeded in 6 well plates for 24 h before drug treatment for a further 24 h. Media was removed and RNA was extracted using an RNeasy Mini Kit with on column DNAse digestion (#74104 and #79254 Qiagen) following the manufacturer’s protocol. RNA quality was assessed using A260/A280 ratio and concentrations normalized. 100 ng of RNA was analyzed by the using an nCounter® PanCancer Pathways Panel (XT-CSO-PATH1-12, NanoString). Gene expression data was analyzed using nSolver software (NanoString) and data was normalized following the NanoString protocol and analyzed using the advanced analysis module to calculate differential ratios between different drug treatments. Data was exported into Cytoscape^42^ and gene networks constructed using the GeneMania app^43^ and functional enrichment was performed using Gene Ontology.^44^

### 4.10 Cancer cell line expression RNAseq analysis

RNAseq datasets (19Q3 release) from 54 breast cancer cell lines from the Cancer Cell Line Expression (broadinstitute.org/ccle) was analyzed for genes of interest. Data is RNAseq TPM (transcripts per million) gene expression data for protein coding genes using RSEM. Data is Log2 transformed, using a pseudo-count of 1. Database was subset for cell lines and genes of interest and overlaid on a boxplot of the expression data for all breast cancer cell lines in the database. Upper and lower “hinges” correspond to the first and third quartiles. Whiskers extend 1.5 × (interquartile range) of the hinge. Data points beyond the end of the whiskers are outliers.

## Declaration of Competing Interest

The authors declare that they have no known competing financial interests or personal relationships that could have appeared to influence the work reported in this paper.
