# Supplementary material for: High content phenotypic screening identifies serotonin receptor modulators with selective activity upon breast cancer cell cycle and cytokine signaling pathways
Source: Bioorg Med Chem. 2020 Jan 1;28(1):115209. doi: 10.1016/j.bmc.2019.115209 (PMC6961118; doi:10.1016/j.bmc.2019.115209)
Supplement: Supplementary Figure 1 [file mmc1.pdf]

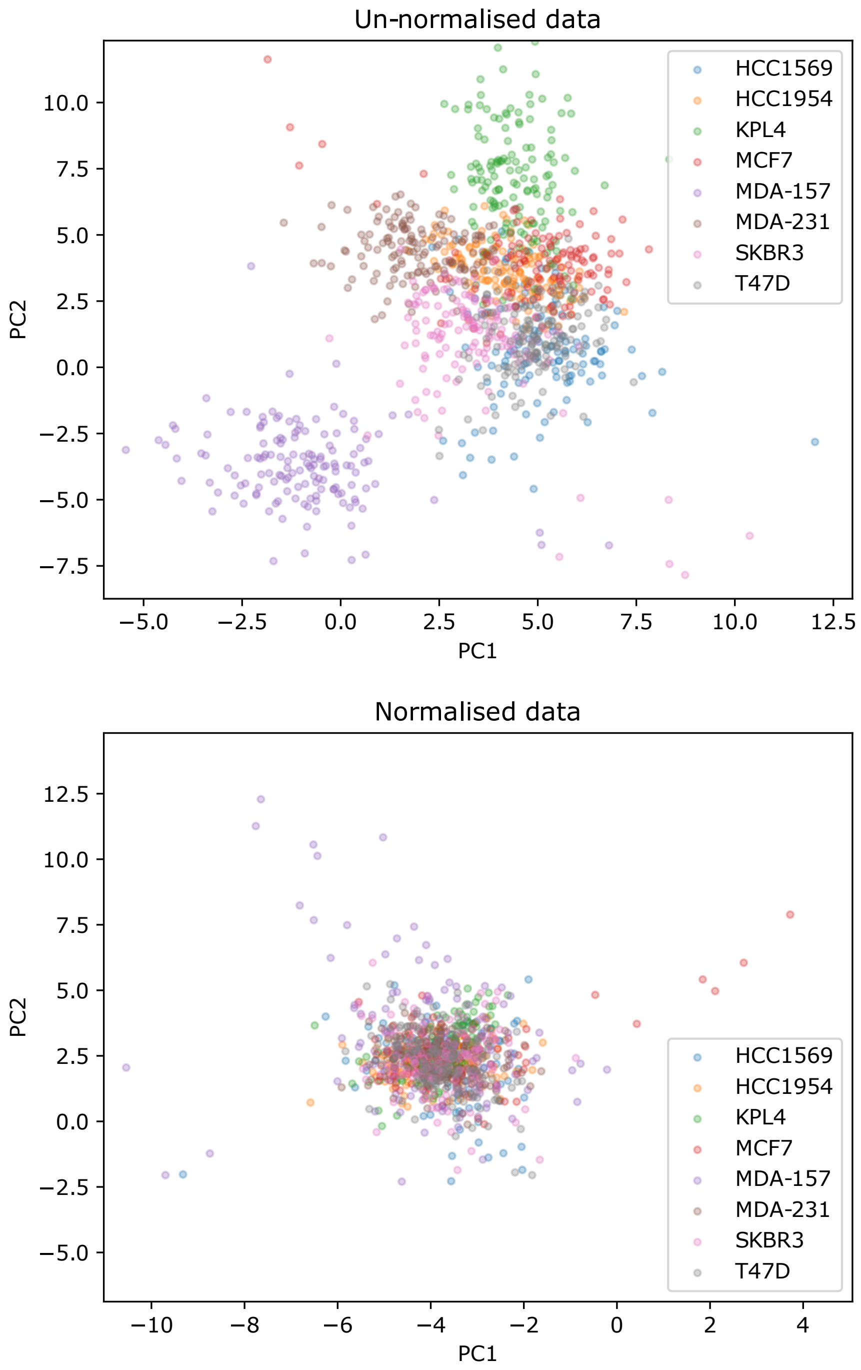

Supplementary Figure S1. Comparison of pre- and post-normalisation of basal cell morphology. Principal component analysis of pre- and post-normalised cell line features demonstrating the removal of inherent cell line morphological differences.
