# Supplementary material for: High content phenotypic screening identifies serotonin receptor modulators with selective activity upon breast cancer cell cycle and cytokine signaling pathways
Source: Bioorg Med Chem. 2020 Jan 1;28(1):115209. doi: 10.1016/j.bmc.2019.115209 (PMC6961118; doi:10.1016/j.bmc.2019.115209)
Supplement: Supplementary Figure 2 [file mmc2.pdf]

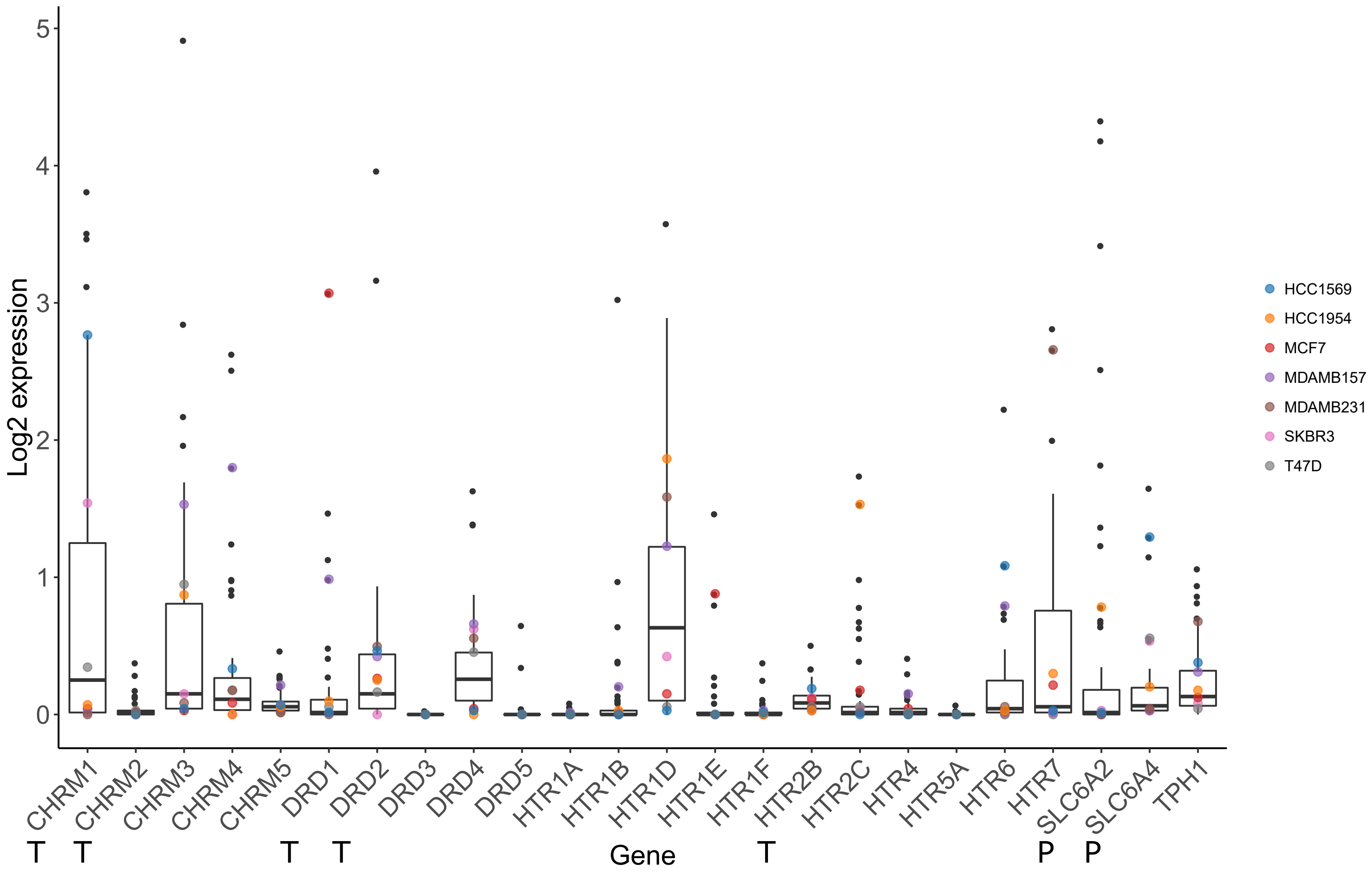


**Supplementary Figure 2**. Compound target gene expression analysis in breast cancer cell lines. RNAseq gene expression data from 72 breast cancer cell lines from the Cancer Cell Line Encyclopedia were analysed for protriptyline (P) and triflupromazine (T) target gene expression. Database was subset for cell lines and genes of interest and overlaid on a boxplot of the expression data for all 72 breast cancer cell lines in the database. Upper and lower "hinges" correspond to the first and third quartiles. Whiskers extend 1.5 × (interquartile range) of the hinge. Data points beyond the end of the whiskers are outliers.
